# ASSOCIATION BETWEEN PERCEIVED CONTROL AND COGNITIVE FUNCTION AMONG STROKE SURVIVORS IN CHINA: A LONGITUDINAL STUDY

**DOI:** 10.1093/geroni/igac059.1850

**Published:** 2022-12-20

**Authors:** Zhijian Liu, Huanzhi Zhu, Jing Wang, Bei Wu, Qin Wang, Juan Li

**Affiliations:** Naval Military Medical University, Shanghai, Shanghai, China (People's Republic); Naval Military Medical University, Shanghai, Shanghai, China (People's Republic); University of North Carolina at Chapel Hill, Chapel Hill, North Carolina, United States; New York University, New York, New York, United States; Linyi People’s Hospital, Linyi, Shandong, China (People's Republic); Huashan Hospital affiliated to Fudan University, Shanghai, Shanghai, China (People's Republic)

## Abstract

This study explored the association between perceived control and cognitive function among stroke survivors in China. We conducted a longitudinal study and assessed perceived control (by Perceived Control in Health Care Questionnaire) and cognitive function (by Montreal Cognitive Assessment, MoCA) of 231 stroke survivors at the acute stage, 3, 6 months after onset from two stroke centers in Shanghai and Linyi from June to December 2020. General linear mixed model was used for analysis. Perceived control was at a moderate level, and the average score of MoCA showed cognitive impairment at 3 waves. Both perceived control and cognitive function improved with time. Perceived control was positively associated with cognitive function (β=0.08, p＜0.001). After controlling for stroke severity, risk factors of cognitive impairment, age, gender, and education, the association was still significant (β=0.04, p＜0.001). These findings suggest that perceived control may be a potential target in cognitive interventions for stroke survivors.

